# ROI-SSIM guided framework for 3D knee MRI reconstruction under artifact degradation

**DOI:** 10.1016/j.mex.2026.104196

**Published:** 2026-10-08

**Authors:** Amanali Bekbolat, Almira Askhatova, Aibek Niyetkaliyev, Prashant Jamwal, Kassymbek Ozhikenov, Zhadyra Alimbayeva, Roza Beisembekova, Yerbolat Igembay

**Affiliations:** a Department of Robotics and Mechatronics, Nazarbayev University, Astana, 010000, Kazakhstan; b Department of Electrical and Computer Engineering, Nazarbayev University, Astana, 010000, Kazakhstan; c Nazarbayev University Research Administration (NURA) Private Institution, Astana, 010000, Kazakhstan; d Department of Robotics and Technical Means of Automation, Satbayev University, Almaty, 050000, Kazakhstan

**Keywords:** Magnetic resonance imaging, Image reconstruction, Transformers, Structural similarity, Artifact robustness, Region-aware loss, Deep learning

## Abstract

Magnetic resonance imaging (MRI) reconstruction is highly sensitive to undersampling and acquisition artifacts. Although deep learning-based approaches have significantly improved reconstruction quality, they are typically optimized using global fidelity metrics that may not fully reflect reconstruction quality within clinically important structures such as the anterior cruciate ligament (ACL), meniscus, and articular cartilage. This study proposes a region-aware MRI reconstruction framework based on a Vision Transformer autoencoder (ViT-AE) guided by an ROI Structural Similarity (ROI-SSIM) loss. The method leverages fastMRI+ bounding-box annotations and incorporates a hybrid artifact simulation framework spanning k-space and image-domain degradations. Experiments on the fastMRI knee single-coil validation set (PD contrast, R=4) show that the proposed ViT-AE achieves reconstruction fidelity competitive with established baselines (SSIM 0.823, Peak Signal-to-Noise Ratio (PSNR) 32.4dB, Normalized Mean Squared Error (NMSE) 0.0147), while ROI-SSIM supervision improves structural fidelity within clinically relevant annotated regions. Under mixed artifact conditions, region-specific structural similarity decreased by only 2.9%, compared with a 6.8% decrease in global SSIM. These findings support the use of region-aware supervision for artifact-robust MRI reconstruction.

Overall, the method presented in this paper:

Introduces ROI-SSIM supervision for region-aware MRI reconstruction.
Develops a hybrid artifact simulation framework spanning k-space and image-domain degradations.
Demonstrates improved anatomical preservation under artifact conditions.

## Graphical abstract

**Figure:**
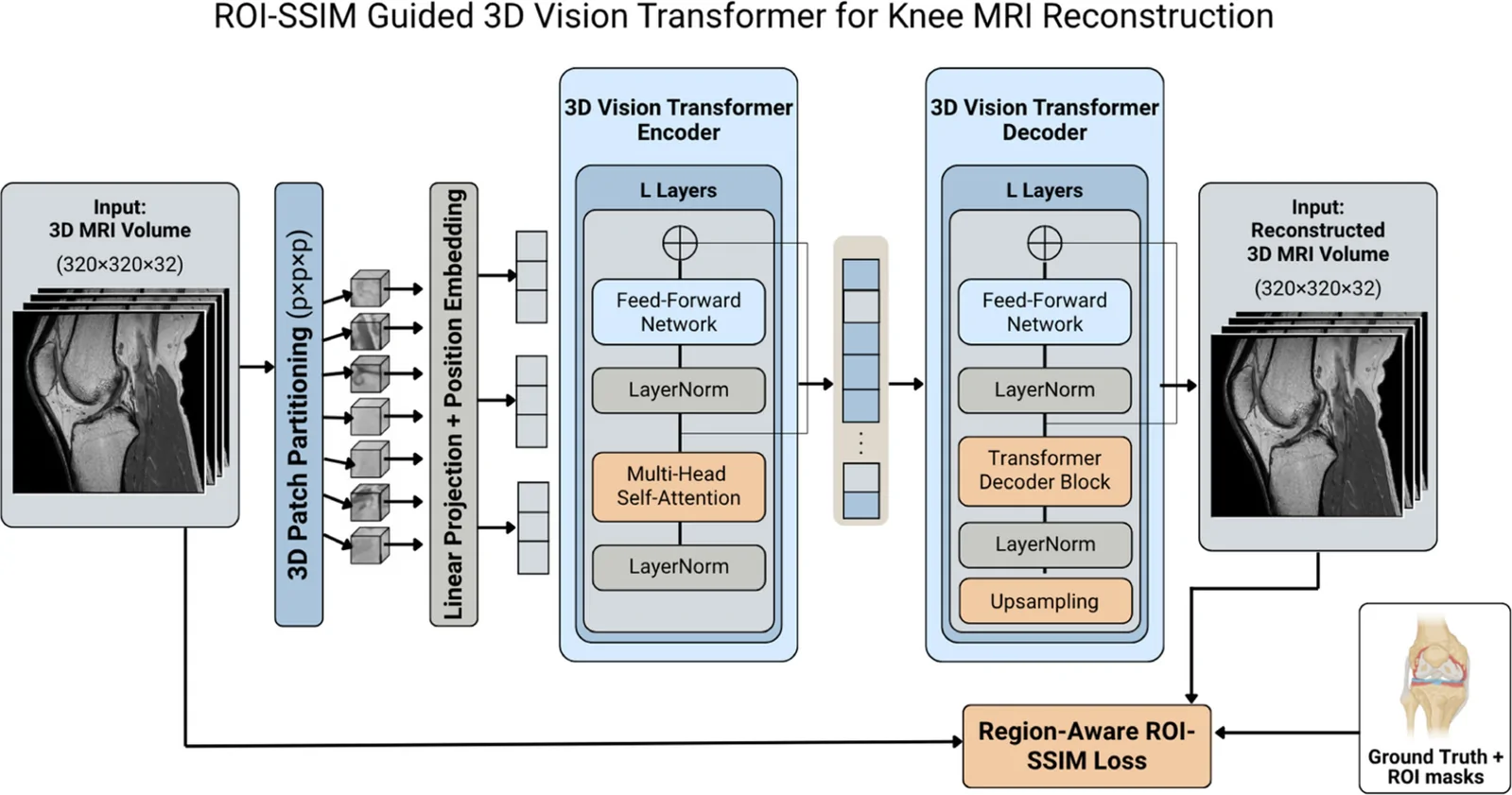

**Specifications table**

**Table:** 

**Subject area**	*Mathematics and Statistics*
**More specific subject area**	Medical Image Reconstruction, Deep Learning for MRI, Region-of-Interest Guided Optimization
**Name of your method**	ROI-SSIM Guided Framework for 3D Knee MRI Reconstruction under Artifact Degradation
**Name and reference of original method**	Modified 3D Vision Transformer Autoencoder (ViT-AE) reconstruction framework incorporating ROI-SSIM supervision and artifact-aware training. Dosovitskiy A. et al. An Image is Worth 16 × 16 Words: Transformers for Image Recognition at Scale. arXiv:2010.11929, 2020; Guo P. et al. ReconFormer: Accelerated MRI Reconstruction Using Recurrent Transformer. IEEE Trans. Med. Imaging, 2024.
**Resource availability**	*Dataset*: fastMRI and fastMRI+ knee MRI datasets (NYU Langone Health): https://fastmri.med.nyu.edu/ Zbontar, J.; Knoll, F.; Sriram, A.; Murrell, T.; Huang, Z.; Muckley, M.J.; Defazio, A.; Stern, R.; Johnson, P.; Bruno, M.; et al. fastMRI: An Open Dataset and Benchmarks for Accelerated MRI. arXiv preprint arXiv:1811.08839, 2018. R. Zhao et al., “fastMRI+, Clinical pathology annotations for knee and brain fully sampled magnetic resonance imaging data,” Sci Data, vol. 9, no. 1, p. 152, Apr. 2022, doi: 10.1038/s41597-022-01255-z.

## Background

Magnetic Resonance Imaging (MRI) is a widely applied clinical method for non-invasive diagnosis of abnormalities, providing high-resolution three-dimensional views of internal anatomy [1]. In this modality, image formation depends on radiofrequency (RF) signal emissions during tissue relaxation, which encode anatomical information [2]. Consequently, this inherent dependence on precise spin alignment and signal acquisition makes MRI particularly sensitive to external disturbances: even slight motion, fluctuations in field homogeneity, or local susceptibility differences can violate the assumptions of spatial encoding and introduce artifacts [3,4].

Recent advances in deep learning have significantly improved MRI reconstruction performance. Early convolutional architectures such as the U-Net [5] and its variants successfully learned end-to-end mappings from zero-filled reconstructions to fully sampled images, establishing a strong baseline for subsequent methods. Variational networks (VarNet) [6,7] and model-based unrolled optimization frameworks further advanced this paradigm by embedding domain-specific priors into the learning process, thereby achieving state-of-the-art performance on large-scale benchmarks such as the fastMRI dataset. More recently, transformer-based architectures [8] and diffusion probabilistic models [9] have demonstrated superior global consistency and generalization, marking a shift from local convolutional operators to global self-attention mechanisms.

A reconstruction may achieve a high global SSIM while failing to preserve the structural integrity of small ligaments or cartilage lesions that are critical for diagnosis. This discrepancy between numerical quality metrics and clinical perception highlights a critical limitation of current evaluation protocols. Moreover, most existing reconstruction networks are trained exclusively on clean, artifact-free data, whereas real-world acquisitions are frequently degraded by motion, Gibbs ringing, partial Fourier truncation, bias field inhomogeneity, and metal-induced distortions.

With the emergence of Vision Transformers (ViTs) [10], self-attention mechanisms enable effective modeling of long-range dependencies in medical imaging. Transformer-based architectures have recently been explored for MRI reconstruction, offering enhanced contextual reasoning across both spatial and slice dimensions. Swin-transformer-based approaches applied to MRI reconstruction leverage hierarchical windowed attention to capture local and global features at multiple resolutions [11]. Similarly, ReconFormer [8] and transformer autoencoders [12] demonstrated improved perceptual quality compared with CNN baselines, particularly in volumetric datasets. Despite these advancements, transformer-based MRI reconstruction models typically employ global loss formulations based on metrics such as SSIM or PSNR averaged over entire volumes [5]. As a result, they may fail to consistently preserve fine-grained anatomical structures, where even minor reconstruction errors can affect diagnosis.

Motivated by these limitations, this study proposes a region-aware MRI reconstruction framework based on a 3D Vision Transformer autoencoder guided by a Region-of-Interest Structural Similarity (ROI-SSIM) loss. By explicitly prioritizing diagnostically relevant anatomical regions and evaluating performance under realistic artifact degradation, the proposed framework aims to improve the clinical relevance and robustness of MRI reconstruction.

## Method details

### Data preparation

Experiments were conducted on the fastMRI and fastMRI+ knee MRI datasets [13,14], which provide fully sampled single-coil and multi-coil acquisitions together with bounding-box annotations for the anterior cruciate ligament (ACL), meniscus, and cartilage. The official fastMRI data splits were used throughout this study, with training, validation, and test partitions defined at the study level to prevent data leakage between subsets. Only the training and validation subsets contain publicly available reference reconstructions and were therefore used for supervised learning and quantitative evaluation.

For preprocessing, single-coil k-space data were reconstructed using a centered two-dimensional inverse Fourier transform with orthonormal scaling. Each volume was center-cropped in-plane to a 320×320 reconstruction frame and resampled along the slice dimension to 32 slices using linear interpolation. The analysis was restricted to CORPD_FBK acquisitions to maintain consistent proton-density contrast across the dataset. Each volume was normalized to the [0, 1] range using the 1st and 99th intensity percentiles of the fully sampled reference volume, with the same normalization constants applied to the corresponding degraded input to maintain a common intensity scale.

For region-aware supervision, anatomy-specific bounding boxes from fastMRI+ were converted into binary volumetric masks $M_{ROI}$ corresponding to the ACL, meniscus, and articular cartilage. Each fastMRI+ annotation was converted into a binary mask at its corresponding slice location. The annotated bounding box was rasterized as an axis-aligned rectangular region in the 320×320 reconstruction frame, with pixels inside the bounding box assigned a value of 1 and all remaining pixels assigned a value of 0. The volumetric mask for each anatomical region was constructed by stacking these slice-level masks. Bounding boxes were not extruded or interpolated across unannotated slices, and no smoothing, dilation, erosion, or other morphological operations were applied at the mask boundaries. When multiple bounding boxes corresponding to the same anatomical region overlapped within a slice, their union was used.

For ROI selection, annotations corresponding to the ACL, meniscus, or articular cartilage were assigned to their respective anatomical regions. Annotations corresponding to other findings, including bone marrow and subchondral edema, subchondral cysts, joint effusion, and posterior cruciate and collateral ligament findings, were excluded from the region-aware objective. The three anatomical regions were maintained as separate masks for region-specific loss computation and evaluation. Volumes without an annotation for a given region did not contribute to that region-specific loss term and were excluded from the corresponding region-specific evaluation metric.

### Artifact simulation framework

To ensure robustness and clinical realism, a hybrid artifact simulation pipeline was developed to emulate degradations in both k-space and image domains. All parameters are randomized per volume, and multiple artifacts may co-occur. The physical motivation and modeling of each artifact are detailed below.

• **Undersampling (k-space)**. MRI acceleration via partial k-space acquisition induces aliasing artifacts. A variable-density Cartesian mask $M(k_{x},\;k_{y})$ with a central coverage fraction $f_{c}\epsilon \;\left[0.08,\;0.15\right]$ and acceleration factor Rϵ[2,6] is applied, in accordance with fastMRI and compressed sensing conventions [13,15]. The undersampled data $K_{u}\;=\;K\;⊙\;M$ are inverse-FFT reconstructed, leading to characteristic folding artifacts.
• **Motion (k-space)**. Patient motion introduces phase inconsistencies across phase-encoding lines. We simulate rigid in-plane motion using sinusoidal phase modulation [3]:
  $$K_{m}\left(k_{x},\;k_{y}\right)\;=\;K\left(k_{x},\;k_{y}\right)\;\cdot \;e^{i\varphi \left(ky\right)},$$
  $$\varphi \left(ky\right)\;=\;\varphi_{max}\;\cdot \;sin\left(2\pi f_{k}y\;+\;\theta \right),$$

where$\;\varphi_{max}\;\epsilon \;\left[0.5,\;2.0\right]$ rad for 10−20% of lines.

• **Partial Fourier (k-space)**. Partial Fourier sampling acquires slightly more than half of k-space and reconstructs missing data by conjugate symmetry. We truncate high-frequency rows, keeping a fraction $f_{p}\;\epsilon \;\left[0.7,\;0.9\right]$, producing edge blurring.
• **Spike Noise (k-space)**. System spikes are modeled by injecting random high-amplitude impulses at sparse k-space locations:
  $$K_{s}\left(k_{x},\;k_{y}\right)=K\left(k_{x},\;k_{y}\right)+\sum_{i=1}^{n_{s}}A_{i}\delta \left(k_{x}-k_{x,i},\;k_{y}-k_{y,i}\right),$$

where $n_{s}\epsilon \;\left[1,3\right]$ is the number of spikes and $A_{i}=\alpha_{i}max\left(\left|K\right|\right),\;\alpha_{i}\;\epsilon \;\left[5,12\right].$ Following inverse Fourier reconstruction, these impulses produce characteristic streaking and herringbone-like artifacts.

• **Zipper Artifact (hybrid)**. Zipper artifacts from periodic RF interference are synthesized by adding narrow sinusoidal bands (width 1–2 px, amplitude 1.2−1.6× the local signal intensity) in vertical or horizontal orientation.
• **Wrap-Around (image)**. Insufficient field-of-view coverage is emulated by circularly shifting the image by 10–20% of its width [16].
• **Partial Volume (image)**. Finite slice thickness causes through-plane averaging; Gaussian blurring with $\sigma_{x-slice}\;\epsilon \;\left[1,\;2\right]$ models this effect [17].
• **Chemical Shift (image)**. Frequency offsets between fat and water create spatial misregistration; we shift fat-dominant regions by 0.5–1.0 px along the frequency-encoding axis [18,19].
• **Gibbs Ringing (image)**. Truncated k-space leads to oscillations near edges. A sinc-windowed frequency cutoff (0.7–0.9) produces realistic ringing [20].
• **Bias-Field Inhomogeneity (image)**. Smooth intensity drift due to B-field non-uniformity is modeled as:
  $$x_{b}\left(x,y\right)\;=\;x\left(x,y\right)\;\cdot \left(1\;+\;b_{\left(x,y\right)}\right),$$

where $b_{\left(x,y\right)}$ is a Gaussian low-frequency field (amplitude 0.2−0.4, σ0.4−0.6 of FOV) [21].

• **Metal Susceptibility (image)**. Magnetic field perturbation near metallic implants is emulated by local phase gradients ∥∇φ∥ϵ[0.01,0.03] rad/pixel within a 30–50 px radius, causing distortion and signal voids [22].
• **Magic-Angle Effect (image)**. Signal anisotropy in collagenous tissues oriented at ≈ 55° to B₀ is modeled by boosting voxel intensity by 30–60% within a sector at $55^{\circ }$ [23].

All artifact parameters are randomized independently for each volume, and multiple artifacts may be applied simultaneously. Representative examples of the simulated artifact degradations are shown in Fig. 1.

**Fig. 1:**
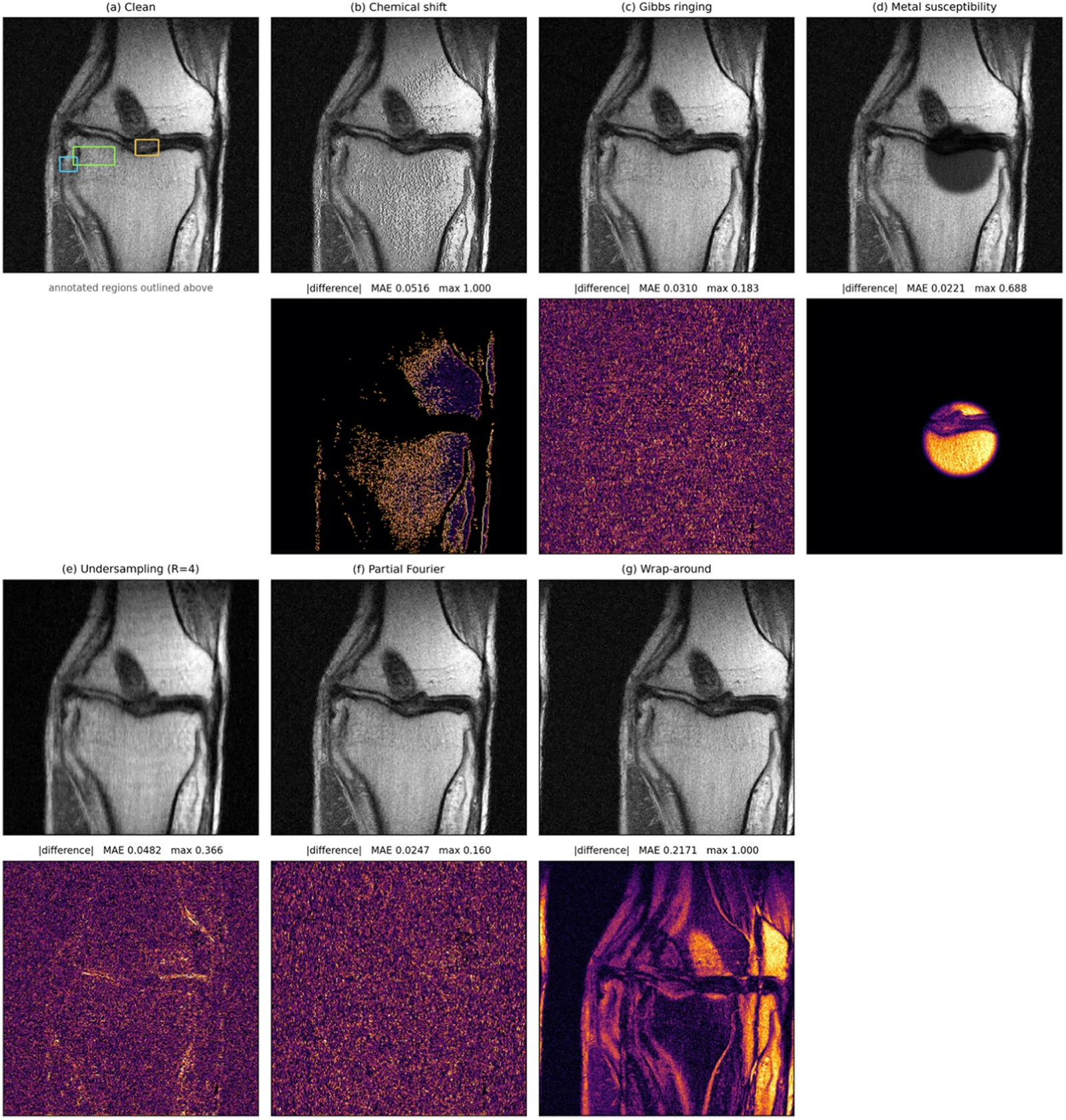
Representative artifact simulations for a single-coil coronal proton-density (PD) knee MRI. (a) Clean reference image with annotated regions of interest; (b) chemical-shift artifact; (c) Gibbs ringing; (d) metal-induced susceptibility artifact; (e) R=4 undersampling; (f) partial-Fourier acquisition; and (g) wrap-around artifact. Absolute difference maps relative to the clean reference are shown below each degraded image, with the corresponding mean absolute error (MAE) and maximum absolute error.

### Region-aware 3D vision transformer autoencoder for knee MRI reconstruction

To address the limitations of 2D convolutional operators in capturing volumetric dependencies, we propose a 3D Vision Transformer Autoencoder (ViT-AE). The architecture is designed to reconstruct a 3D volume $V\;\epsilon \;R^{H\times W\times D}$ from zero-filled reconstructions obtained from undersampled and artifact-corrupted k-space data (Fig. 2). The proposed model consists of the following key components:

1. *3D Patch Partitioning*. The input volume is divided into non-overlapping 3D patches of size p×p×p. Each patch is flattened into a vector and projected through a linear layer into a d-dimensional embedding space.
2. *Volumetric Self-Attention*. The encoder consists of L transformer blocks. Each block employs Multi-Head Self-Attention (MHSA) to model global spatial relationships across the entire knee volume. This enables the model to leverage anatomical continuity across adjacent and distant slices to resolve local reconstruction artifacts.
3. *Latent Bottleneck and Reconstruction*. The decoder mirrors the encoder architecture and employs transformer blocks together with progressive upsampling layers to reconstruct the volume at its original resolution (320×320×32).

**Fig. 2:**
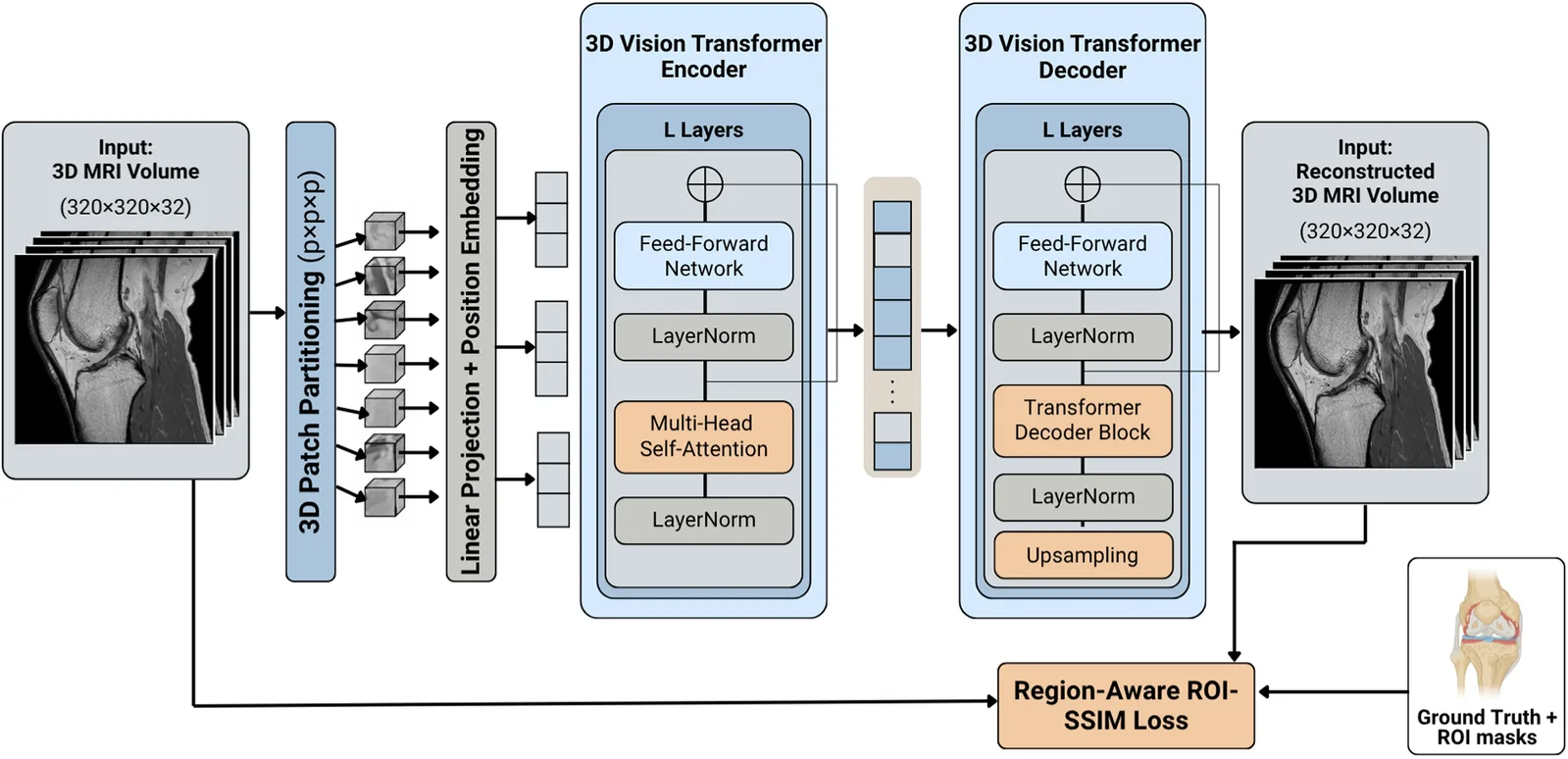
Region-aware 3D Vision Transformer Autoencoder (ViT-AE) architecture for knee MRI reconstruction. ROI-SSIM supervision is applied using ACL, meniscus, and cartilage region masks to prioritize diagnostically relevant anatomical structures during reconstruction.

### Region-of-interest structural similarity loss

A central component of the proposed framework is the integration of ROI-guided supervision into the reconstruction process. ROI masks corresponding to the ACL, meniscus, and cartilage were generated from fastMRI+ annotations and used for region-aware supervision [13,14] (Fig. 3).

**Fig. 3:**
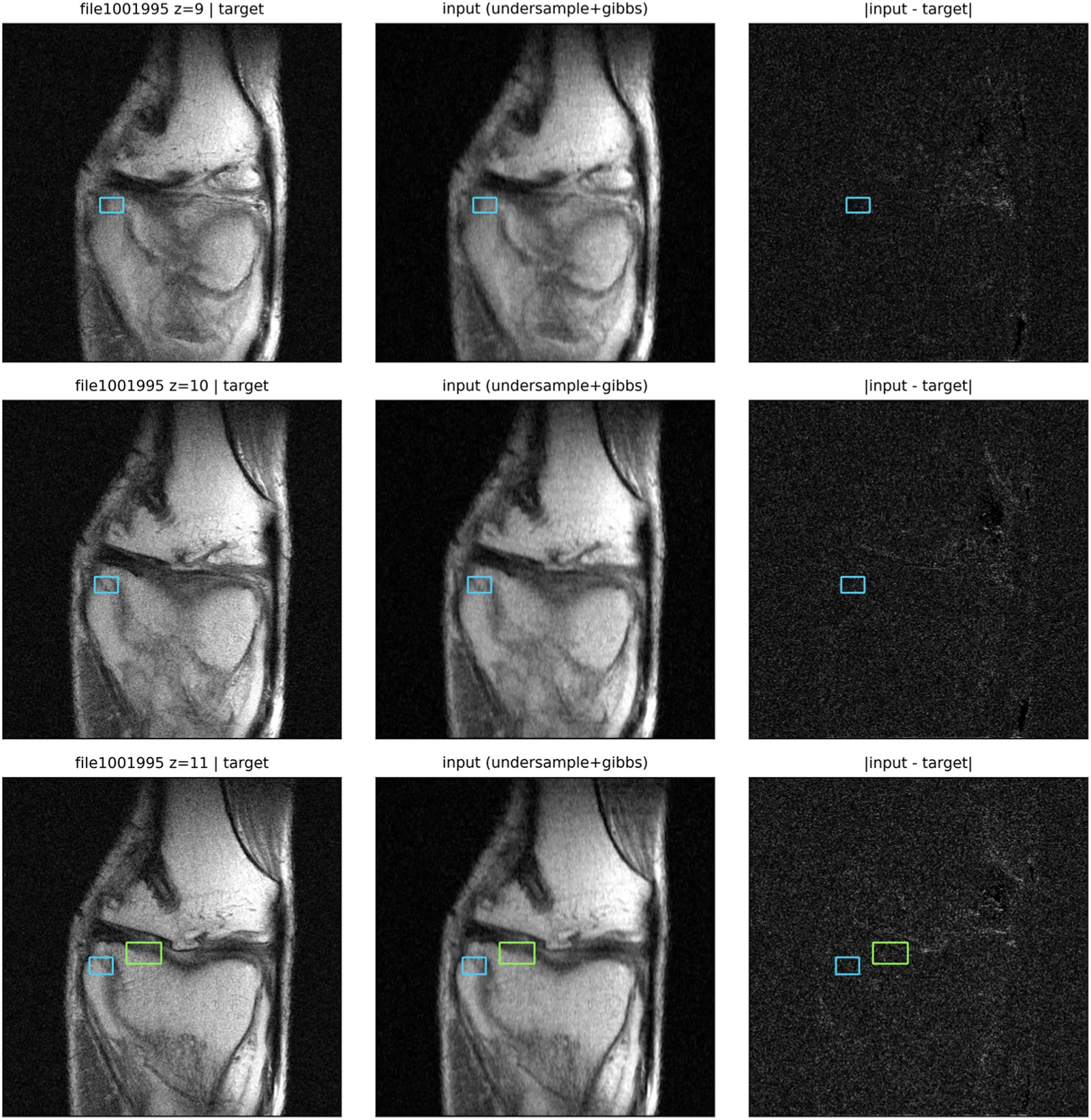
Ground-truth knee MRI slice with fastMRI+ annotations used to generate volumetric ROI masks for ACL, meniscus, and cartilage supervision.

The total objective function $L_{total}$ is defined as a weighted combination of global and regional fidelity terms:

$$L_{total}\;=\;\alpha L1\;+\;\beta L_{Global-SSIM}\;+\;\gamma L_{ROI-SSIM},$$

where the Region-of-Interest Structural Similarity (ROI-SSIM) loss is formulated as

$$L_{ROI-SSIM}\;=\;\sum_{r\epsilon \left\{ACL,Men.,Cart.\right\}}w_{r}\left(1\;-\;SSIM\left(G\;⊙\;Mr,\;\hat{G}\;⊙\;Mr\right)\right),\;$$

Here, G and $\hat{G}$ represent the ground-truth and reconstructed volumes, respectively; ⊙ denotes the Hadamard (element-wise) product used to isolate specific anatomical regions; and $w_{r}$ are region-specific weighting coefficients. In all experiments, α=1.0,β=1.0,γ=2.0, and$\;w_{r}\;=\;1.0$ for all three anatomical regions. These coefficients were specified a priori and were not tuned on validation performance; no alternative coefficient configuration was trained. Setting α = β= 1.0 assigns equal weighting to the global L₁ and SSIM terms, while γ=2.0 was selected as a predefined design choice to give greater emphasis to structural fidelity within the annotated regions. Equal region weights $w_{r}=1.0$ were used so that no anatomical region among the ACL, meniscus, and cartilage was preferentially weighted. These coefficients were kept unchanged across all experiments to maintain a consistent optimization objective.

ROI-SSIM was computed using an 11 × 11 local SSIM window. Consequently, for pixels near ROI boundaries, the local SSIM calculation may incorporate neighboring pixels outside the annotated bounding box, although the loss itself is evaluated using the corresponding binary ROI mask. SSIM was chosen as the basis for region-aware supervision due to its sensitivity to local structural contrast and luminance patterns, which correlate more closely with radiological perception than pixel-wise losses such as L1 or L2 [24]. Unlike perceptual losses based on pretrained networks, SSIM is modality-agnostic, does not rely on external training data, and is commonly used in MRI reconstruction benchmarks, ensuring compatibility with established evaluation protocols.

## Method validation

### Experimental setup and evaluation protocol

Quantitative evaluation was restricted to the validation subset because ground-truth reconstructions for the test set are not publicly available. Experiments were conducted under the R=4 Cartesian undersampling configuration, the most commonly reported setting in the fastMRI literature. Zero-filled inverse Fourier reconstructions served as network inputs.

Reconstruction quality was evaluated using SSIM, PSNR, and NMSE, computed according to the fastMRI evaluation protocol. Metrics were calculated slice-wise and averaged at the volume level. Region-specific fidelity was assessed using the proposed ROI-SSIM, computed within annotated anatomical regions. Unless otherwise stated, reported results correspond to the mean over the validation set. Baseline results were obtained from published validation-set evaluations using the fastMRI knee dataset under the R=4 acceleration setting. No test-server submissions were used. For classical baselines, we report the best-performing configurations under the R=4 setting as provided in the fastMRI benchmark study [13]. Models were trained using AdamW with an initial learning rate of $2\cdot 10^{-4}$, weight decay of $5\cdot 10^{-2}$, and β=0.9,0.95. A cosine learning-rate decay schedule was applied over 200 epochs, with a batch size of 4. Gradient norms were clipped at 1.0, and mixed-precision training was used. The output convolution was zero-initialized, and the predicted residual was added to the zero-filled input, such that the initial reconstruction approximated the identity mapping. This residual formulation was used to improve optimization stability during early training. Model checkpoints were selected according to validation ROI-SSIM. All experiments were implemented in Python 3.11 using PyTorch 2.10.0 with CUDA 13.0 and were performed on a single NVIDIA A100 80 GB GPU.

### Computational cost

The proposed 3D ViT-AE contains approximately 47.9M trainable parameters, compared with 53.54M for the U-Net baseline. Training converged in approximately 22 GPU-hours on a single NVIDIA A100 80 GB GPU. Inference time for a full 320×320×32 volume is approximately 135 ms, versus 12 ms for U-Net. The increased computational cost is primarily attributable to volumetric self-attention, which is known to incur substantially higher computational and memory complexity than convolutional operators. Strategies such as windowed attention [10,11] or mixed-precision inference could reduce latency in deployment settings and are left as future work. The increased computational requirements represent a trade-off between efficiency and regional reconstruction fidelity. Although the proposed transformer-based model has substantially higher inference latency than the U-Net baseline (135 ms vs. 12 ms per volume), the absolute difference of approximately 123 ms remains small relative to MRI acquisition times. This additional cost may be justified in applications where preservation of diagnostically relevant structures is prioritized, particularly ligamentous and cartilaginous regions for which the proposed method demonstrated improved region-specific reconstruction fidelity. Conversely, lighter convolutional architectures may be preferable in high-throughput or resource-constrained settings where computational efficiency is the primary consideration.

### Quantitative results on clean validation data

Table 1 reports quantitative reconstruction performance on the fastMRI validation set under the R=4 undersampling condition without additional simulated artifacts. Among the listed methods, the proposed ViT-AE achieves the highest SSIM, although its PSNR is lower than that of the published U-Net baseline. Compared with the baseline ViT-AE trained using only global loss functions, the incorporation of ROI-guided supervision produces a modest improvement in global SSIM. Specifically, ROI-L₁ increases SSIM from 0.819 to 0.820, while ROI-SSIM further improves SSIM to 0.823. These results indicate that emphasizing diagnostically relevant anatomical regions during training does not degrade overall reconstruction quality and may provide measurable benefits in structural fidelity. Among the proposed variants, ROI-SSIM achieves the highest global SSIM while maintaining comparable PSNR and NMSE values. The consistent improvement over ROI-L₁ suggests that structural similarity is a more effective region-aware supervision signal than pixel-wise regional penalties alone, supporting the use of perceptual region-based objectives for MRI reconstruction.

**Table 1:** Validation-set performance on fastMRI knee single-coil reconstruction. † indicates results reported in the original fastMRI benchmark study. ‡ indicates single-coil fastMRI results reported by Guo et al. [8]. Published baselines were not retrained in this study. All proposed variants are trained and evaluated under identical settings, without artifact augmentation; the artifact-augmented model is reported in Table 3.

Method	Params	SSIM ↑	PSNR ↑ (dB)	NMSE ↓
*Published single-coil validation baselines*				
Classical TV (BART)† [13]	N/A	0.645	31.4	0.0287
U-Net-128† [25]	53.54 M	0.815	34.01	0.0154
*Published single-coil fastMRI reference*				
ReconFormer‡ [8]	1.141 M	0.7383	32.73	0.0310
*Proposed variants*				
ViT-AE (global loss only)	47.9 M	0.819	32.5	0.0145
ViT-AE + ROI-L₁	47.9 M	0.820	32.3	0.0152
**ViT-AE + ROI-SSIM (proposed)**	**47.9 M**	**0.823**	**32.4**	**0.0147**

For context, Table 1 includes results from established reconstruction methods. These values were taken from the cited studies rather than reproduced under the present training pipeline, and the global metrics do not rank the methods consistently: the proposed model attains a higher SSIM than the published U-Net baseline (0.823 vs. 0.815) but a lower PSNR (32.4 vs. 34.01 dB). Global metrics alone are therefore insufficient to establish the benefit of region-aware supervision; they average reconstruction quality over the entire image volume and may underrepresent changes within small but diagnostically important anatomical structures. Accordingly, the effect of ROI-SSIM is assessed below through the controlled comparison with the global-loss ViT-AE, followed by analyses of region-specific fidelity and robustness under artifact degradation.

The effect of ROI-SSIM is more appropriately assessed by comparison with the global-loss ViT-AE, which shares the same architecture and training protocol. Introducing ROI-SSIM increases global SSIM from 0.819 to 0.823, while PSNR changes from 32.5 to 32.4 dB and NMSE from 0.0145 to 0.0147. Thus, the regional term produces a measurable improvement in structural similarity with only minor changes in global pixel-wise fidelity metrics.

### Regional fidelity analysis

To assess localized reconstruction performance, ROI-SSIM was computed independently for the ACL, meniscus, and articular cartilage using the bounding-box masks from fastMRI+. Table 2 reports ROI-SSIM values for the global-loss baseline (ViT-AE without ROI supervision) and the full proposed model (ViT-AE + ROI-SSIM). Results are presented as mean ± standard deviation over the validation volumes containing an annotation for each structure.

**Table 2:** ROI-SSIM within fastMRI+ annotated regions corresponding to ACL, meniscus, and articular cartilage. Results are mean ± standard deviation over validation volumes containing at least one annotation for the corresponding structure. N denotes the number of volumes included in each structure-specific analysis. The ACL comparison (*N* = 14) is under-powered relative to the meniscus (*N* = 53) and cartilage (*N* = 43) comparisons and should be interpreted with caution. *Statistically significant improvement over the global-loss baseline (*p* < 0.01, paired two-sided Wilcoxon signed-rank test performed at the volume level, with predictions paired by volume identifier).

Structure	N (volumes tested)	Annotated slices	ViT-AE (global loss)	ViT-AE + ROI-SSIM	Δ ROI-SSIM
ACL	14	87	0.742 ± 0.031	0.779 ± 0.028	+4.9% *
Meniscus	53	336	0.764 ± 0.024	0.799 ± 0.021	+4.6% *
Cartilage	43	203	0.779 ± 0.019	0.813 ± 0.018	+4.4% *
**Mean (all ROIs)**	**-**	**-**	**0.762 ± 0.025**	**0.797 ± 0.022**	+4.6% *

Across all three anatomical structures, ROI-SSIM supervision yields consistent improvements in region-specific structural similarity. All three improvements are statistically significant under a paired Wilcoxon signed-rank test (*p* < 0.01). The largest gain is observed for the ACL (+4.9%), but this estimate should be interpreted with caution: it rests on only 14 volumes, substantially fewer than the meniscus (*N* = 53) and cartilage (*N* = 43) comparisons, so the ACL test has considerably lower statistical power and the size of the ACL effect is less certain. The meniscus (+4.6%) and cartilage (+4.4%) gains, which are based on larger samples, therefore provide the more reliable evidence of the regional benefit.

Notably, these regional improvements are observed despite only modest changes in global SSIM (<1%), highlighting the limitations of global fidelity metrics in capturing reconstruction quality within clinically relevant anatomical regions. These findings support the use of region-aware supervision for preserving diagnostically relevant structures during MRI reconstruction.

### Ablation study

To isolate the contribution of each design choice, we conduct a controlled ablation study. Table 3 evaluates five variants of the proposed framework, each differing from the preceding row in a single component: (i) the dimensionality of self-attention (2D slice-wise vs. 3D volumetric), (ii) the loss formulation (global only, ROI-L₁, or ROI-SSIM), and (iii) the training data distribution (clean only vs. artifact-augmented). Except for the attention dimensionality being evaluated, all variants were trained using identical optimization settings and evaluation procedures.

**Table 3:** Ablation study on the fastMRI knee validation set (single-coil, R = 4). Each row differs from the previous one in a single component. Global SSIM and mean ROI-SSIM (averaged over ACL, meniscus, cartilage) are reported.

Variant	3D Attention	Region-Aware Loss	SSIM ↑	Mean ROI-SSIM ↑
2D slice ViT-AE (global loss)	✗	✗	0.804	0.751
3D ViT-AE (global loss)	✓	✗	0.819	0.762
3D ViT-AE + ROI-L₁ (no artifact aug.)	✓	✓	0.820	0.774
3D ViT-AE + ROI-SSIM (no artifact aug.)	✓	✓	0.823	0.797
**3D ViT-AE + ROI-SSIM + artifact aug. (full)**	**✓**	**✓**	**0.825**	**0.806**

The ablation results reveal several consistent trends. First, replacing 2D slice-wise attention with 3D volumetric self-attention increases global SSIM from 0.804 to 0.819 and mean ROI-SSIM from 0.751 to 0.762, indicating that modeling inter-slice dependencies is beneficial for musculoskeletal structures with strong anatomical continuity across slices. Second, the addition of ROI-SSIM supervision provides the largest improvement in regional fidelity, increasing mean ROI-SSIM from 0.762 to 0.797 while only modestly affecting global SSIM. Third, artifact-augmented training adds a further +1.1% in mean ROI-SSIM (0.797 to 0.806), consistent with the robustness results in Table 4, and suggests that exposure to realistic degradations during training improves the model's structural preservation under clean conditions as well. Together, these results demonstrate that volumetric attention, ROI-guided supervision, and artifact augmentation each contribute measurable improvements to reconstruction performance, with the largest gains observed in region-specific structural fidelity. These quantitative improvements are also reflected qualitatively in the reconstruction error maps shown in Fig. 4. Compared with the global-loss baseline, ROI-SSIM-guided reconstruction exhibits reduced reconstruction errors within ligamentous and cartilaginous regions, indicating improved preservation of fine anatomical structures.

**Table 4:** Robustness evaluation on the fastMRI knee validation set under simulated artifact degradation. Values represent relative decreases in SSIM and ROI-SSIM compared with clean validation performance.

Artifact Type	Global SSIM ↓	ROI-SSIM ↓
Undersampling	−3.1%	−1.8%
Motion (k-space)	−4.6%	−2.5%
Gibbs ringing	−3.8%	−2.1%
Partial Fourier	−4.2%	−2.7%
Zipper artifact	−2.9%	−1.6%
Bias-field inhomogeneity	−3.4%	−1.9%
Metal susceptibility	−5.3%	−3.2%
**Mixed artifacts**	**−6.8%**	**−2.9%**

**Fig. 4:**
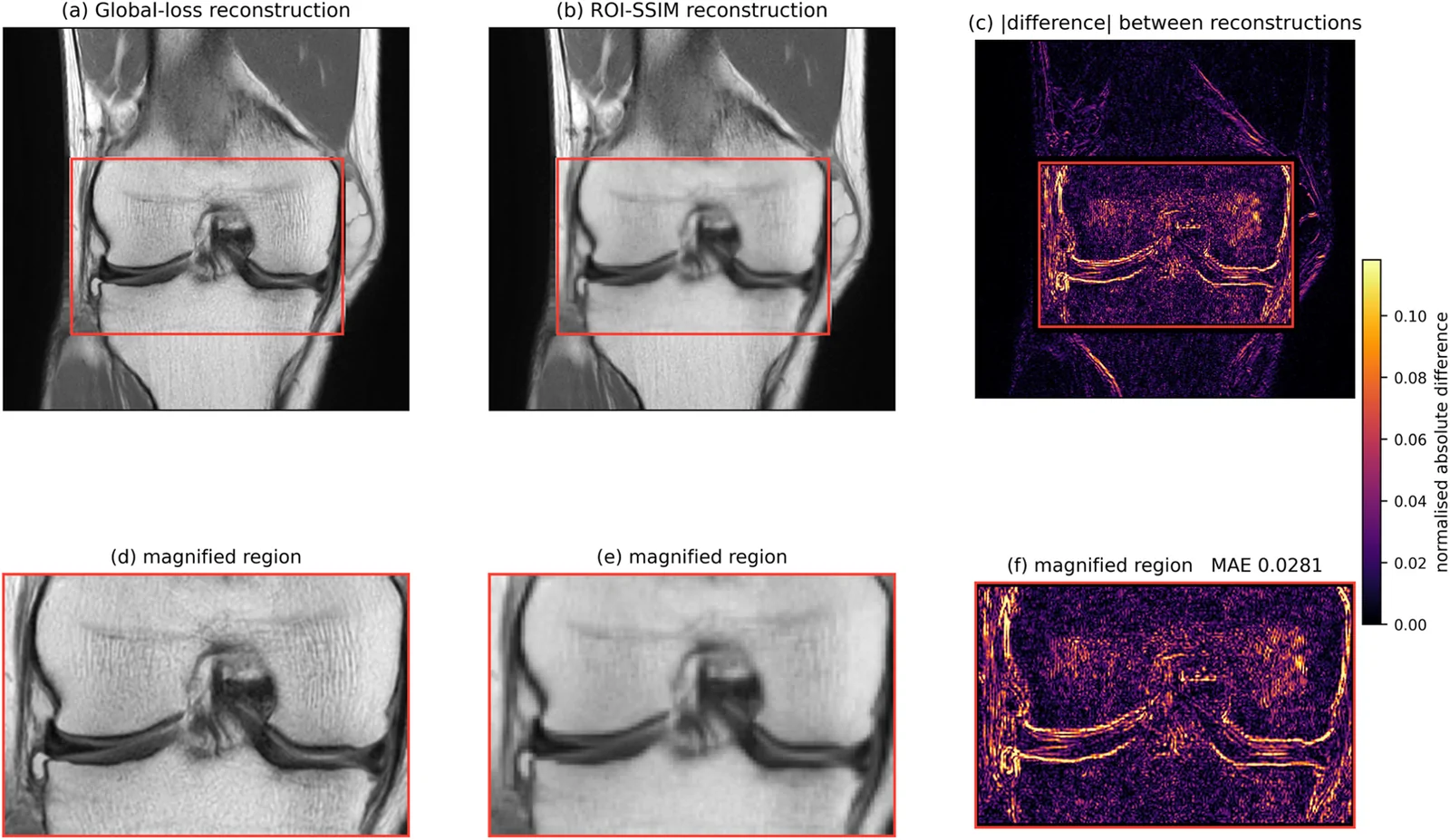
Qualitative comparison of global-loss and ROI-SSIM-guided knee MRI reconstructions.

### Robustness evaluation under artifact degradation

To evaluate robustness under realistic acquisition imperfections, we assess reconstruction performance under controlled artifact perturbations using the hybrid simulation framework. Validation volumes are corrupted by individual artifact types and by mixed degradation, while ground-truth reconstructions remain unchanged.

Artifacts are applied at randomly sampled intensities within clinically plausible ranges and include undersampling irregularities, motion-induced phase errors, Gibbs ringing, partial Fourier truncation, zipper artifacts, bias-field inhomogeneity, and metal susceptibility distortions. For each artifact type, performance is evaluated using both global SSIM and ROI-SSIM, allowing simultaneous assessment of overall reconstruction stability and localized anatomical preservation.

Robustness is quantified using the relative degradation metric, $\Delta SSIM\;=\;SSIM_{clean}\;-\;SSIM_{artifact}\Delta SSIM\;=\;\frac{SSIM_{artifact}\;-\;SSIM_{clean}}{SSIM_{clean}}\;\times \;100\%$, with an analogous definition for ROI-SSIM. Values closer to zero indicate higher robustness. All values report the mean relative degradation with respect to clean validation performance.

Across all evaluated artifact types, the proposed region-aware 3D ViT-AE exhibits consistently lower degradation in ROI-SSIM compared to global SSIM, indicating enhanced resilience within diagnostically relevant anatomical regions. While global reconstruction quality degrades noticeably under severe distortions—particularly in the presence of motion and metal-induced artifacts—the proposed framework preserves structural integrity within the ACL, meniscus, and cartilage to a substantially greater extent. Notably, the largest discrepancy between global and regional degradation is observed under mixed artifact conditions, where multiple distortions co-occur. In this scenario, global SSIM drops by 6.8%, whereas ROI-SSIM degrades by only 2.9%, highlighting the effectiveness of region-aware supervision in stabilizing reconstruction quality under compounded acquisition imperfections. These results suggest that ROI-SSIM optimization encourages the model to prioritize reconstruction fidelity within anatomically meaningful regions, thereby improving robustness within anatomical regions relevant to diagnostic assessment. In contrast, global metrics weight all image regions equally and may therefore be less sensitive to reconstruction quality within diagnostically relevant anatomical structures.

### Practical implications

Taken together, these results indicate the situations in which ROI-guided optimization is expected to offer the greatest advantage over conventional global optimization. The first is when the clinical question concerns small structures that occupy only a minor fraction of the imaged volume, such as the ACL, menisci, and articular cartilage. A global objective is dominated by large regions of bone, fat, and background, so errors confined to these structures contribute little to the loss; this is reflected in the 4.4–4.9% regional gains obtained with a change of less than 1% in global SSIM. The second is when acquisitions are affected by compounded degradations, for example motion in patients who cannot remain still, or susceptibility distortion in postoperative knees with metallic fixation hardware, where the gap between global and regional degradation in Table 4 was largest. The third is when the reconstruction feeds a region-level downstream task, such as automated lesion detection, segmentation, or quantitative cartilage assessment, whose accuracy depends on fidelity within the target structure rather than across the whole field of view. Conversely, the advantage is expected to be smaller when the diagnostic finding is not confined to the annotated structures (for example bone marrow edema or joint effusion, which were excluded from the region-aware objective), when reliable region annotations are unavailable, or when degradation is mild enough that global optimization already preserves the structures of interest. Whether the benefit grows at higher acceleration factors was not examined here, as the evaluation was restricted to R = 4.

### Limitations

Several limitations of the proposed framework should be acknowledged. First, region-aware supervision relies on bounding-box annotations provided by the fastMRI+ dataset. While bounding boxes offer a scalable and practical form of anatomical localization, they provide coarser supervision than pixel-level segmentations and inevitably include surrounding tissues such as fat, bone marrow, and joint fluid. Furthermore, because SSIM is computed using an 11 × 11 local window, pixels near the ROI boundary may also be influenced by neighboring pixels outside the bounding box. Future work could investigate more selective supervision using weak or semi-automatic segmentation, eroded bounding boxes, or distance-weighted masks that concentrate the regional loss toward the target structure.

Second, the experimental evaluation is limited to knee MRI and the single-coil reconstruction setting. Further validation is required to assess generalizability to other anatomical regions, scanners, and acquisition protocols. Extension to multi-coil acquisition would require multi-coil k-space handling, incorporation or estimation of coil sensitivity maps, and sensitivity-aware data consistency, while the region-aware loss could continue to be applied to the reconstructed magnitude image. Extension to other anatomical regions would primarily require appropriate region annotations and retraining on the target anatomy; the ROI-SSIM formulation itself can be retained, although preprocessing, ROI definitions, and model parameters may require adaptation to the target anatomy and acquisition protocol. Whole-volume PSNR, NMSE, and SSIM do not provide a direct assessment of reconstruction quality outside the annotated regions. A separate ROI/non-ROI error decomposition was not performed; therefore, the present results cannot exclude localized high-frequency noise, intensity bias, or other reconstruction changes in non-ROI regions. Future work should explicitly quantify reconstruction fidelity inside and outside annotated regions to determine whether gains in regional structural preservation introduce any localized trade-offs elsewhere in the image.

The present evaluation is also restricted to a homogeneous coronal proton-density knee MRI acquisition subset and does not assess generalization across scanner vendors, field strengths, coil configurations, or sequence parameters. Although percentile-based intensity normalization reduces sensitivity to absolute signal scaling, substantial changes in image contrast may alter the relative appearance of anatomical structures and affect reconstruction performance. Similarly, the artifact-simulation parameter ranges used in this study may require recalibration when transferring the framework to different scanners, field strengths, or acquisition protocols. Because the analysis was restricted to CORPD_FBK acquisitions, generalization across different contrast weightings remains to be established experimentally. In particular, the fastMRI knee data were acquired at a single institution on scanners from a single vendor, so the reported gains have not yet been confirmed on independent sites. Future work should therefore validate the framework on multi-vendor, multi-site data spanning 1.5 T and 3 T field strengths, different receive-coil configurations, and both 2D and 3D sequences, including publicly available raw k-space knee datasets acquired on other vendor platforms, such as SKM-TEA [26], which also provides bounding-box pathology annotations compatible with the present region-aware objective, and prospectively acquired clinical scans. Global and regional metrics should be reported separately for each scanner and protocol, so that any protocol-dependent reduction in the regional benefit can be identified and addressed through fine-tuning or recalibration of the artifact-simulation parameter ranges.

Third, artifact degradations are simulated rather than derived from prospectively acquired corrupted scans. While the simulation framework is grounded in established physical models and clinically plausible parameter ranges, it cannot fully capture the variability encountered in routine clinical practice. Evaluation on prospectively acquired artifact-corrupted data would therefore provide stronger evidence of clinical robustness.

Finally, the weighting coefficients between the global and region-specific loss components were fixed a priori throughout the study and were not selected through validation-set hyperparameter optimization. No sensitivity analysis over the loss coefficients was performed; consequently, the reported configuration should be interpreted as a predefined design choice rather than an empirically optimized setting. The appropriate balance between global and regional supervision may depend on image normalization, ROI size and distribution, anatomical target, degradation severity, and acquisition protocol. Systematic sensitivity analysis and adaptive or dataset-specific weighting strategies should therefore be investigated in future work.

## Ethics statements

This study used the publicly available fastMRI dataset provided by NYU Langone Health [13]. The dataset consists of fully de-identified MRI data collected under institutional review board (IRB) approval and in compliance with applicable data protection regulations. No new data involving human participants were collected, and no additional ethical approval or informed consent was required.

## Declaration of competing interest

The authors declare that they have no known competing financial interests or personal relationships that could have appeared to influence the work reported in this paper.

## Data Availability

Data will be made available on request.
